# Imbalance in Sirt1 Alternative Splicing in Response to Chronic Stress during the Adolescence Period in Female Mice

**DOI:** 10.3390/ijms23094945

**Published:** 2022-04-29

**Authors:** Shir Shlomi, Roni Toledano, Keren Nitzan, Sigal Dror Shahaf, Emanuela P. Break, Dan Frenkel, Ravid Doron

**Affiliations:** 1 Department of Neurobiology, The George S. Wise Faculty of Life Sciences, School of Neurobiology, Biochemistry and Biophysics, Tel Aviv University, Tel Aviv 6997801, Israel; shirshlomi7@gmail.com (S.S.); shahaf.sigal@gmail.com (S.D.S.); dfrenkel@tauex.tau.ac.il (D.F.); 2 Sagol School of Neuroscience, Tel Aviv University, Tel Aviv 6997801, Israel; 3 Department of Education and Psychology, The Open University, Raanana 43107, Israel; Ronitol1988@gmail.com (R.T.); kfridel@gmail.com (K.N.); 321emanuela@gmail.com (E.P.B.)

**Keywords:** unpredictable stress, cognition, Sirt1, TrkB, senescence

## Abstract

Stressful unpredictable life events have been implicated in numerous diseases. It is now becoming clear that some life periods are more vulnerable than others. As adolescence is a sensitive period in brain development, the long-term effects of stress during this period could be significant. We investigated the long-term effects of exposure to unpredictable chronic mild stress in adolescent mice on alternative splicing of Sirtuin 1. One-month-old mice were exposed to 4 weeks of UCMS and examined for anxiety and cognition at the age of 2, 4 and 6 months. We found a rise in anxious behavior immediately after the exposure to stress. Notably, there was a long-term impairment of performance in cognitive tasks and an imbalance in Sirtuin 1 and TrkB receptor alternative splicing in the stress-exposed mice compared with controls. To conclude, our results show that exposure to unpredictable chronic mild stress during adolescence affects cognition in adulthood. Understanding pathways affiliated with stress may help minimize the long-term emotional effects of an unpredictable, stressful event.

## 1. Introduction

Stress is an integral part of modern life. The healthy stress response is aimed at maintaining homeostasis when faced with psychological or physiological challenges. However, chronic or unpredictable stressful events or exposure to stress at critical developmental stages can be maladaptive and dysregulates this homeostasis [1]. Stressful life events have been implicated in the development of cardiovascular diseases [2], cancer [3] and neurodegenerative disease that are affiliated with cognition, such as Alzheimer’s disease [4]. In animal models, chronic stress in adulthood has been associated with cognitive impairments [5,6,7,8], neuronal modifications [6,7,9,10,11] and accelerated cellular senescence [12] and thus could be implicated as a risk factor for age-related cognitive decline. Chronic stress situations induce high-fat food consumption [13]. High-fat diet has been related to cognitive decline [14], and in addition to the exposure to stress, this may yield long-term effects. In fact, recent studies suggest that young children and adolescents are more prone to adopt an unhealthy diet due to the lack of a normal routine. [15]. 

Over the past years, it is becoming clearer that some stages of life are more vulnerable to stress—specifically early childhood and adolescence [16]. In humans, early life stress increases the risk for psychopathology in adulthood [17], impairs the immune system and causes immunosenescence [18]. It is not surprising, then, that when looking at the long-term effect of stress, the majority of the animal studies focused on early-childhood stress exposure [19]. Indeed, early-life stress causes significant impairment in hippocampal development and cognitive deficiencies in adulthood [20]. Moreover, female mice, but not males, exposed to maternal separation during early childhood exhibited increased anxiety [21]. While there is no doubt that early childhood is important, the adolescence period may be particularly susceptible to stress-related neurological changes.

During adolescence, the brain undergoes neurological and biochemical changes. The prefrontal cortex (PFC) undergoes prominent neural alterations [22]. There are also changes in the amygdala and (hypothalamic–pituitary–adrenal) HPA axis [23], which are intrinsically connected to the stress response. These changes make the adolescence period susceptible to psychiatric conditions, such as schizophrenia [24] and major depression [25]. Exposure to stress during adolescence may have different effects on males and females and is also dependent on the severity and the duration of the stressors [26,27].

Here, we sought to investigate the long-term effect on cognition of exposure to unpredictable chronic mild stress during this sensitive age using the ‘unpredictable chronic mild stress’ (UCMS) manipulation on adolescent mice.

UCMS [28] is a well-established method that uses six different physical and social stressors administered randomly. We have previously found that following UCMS, adolescent mice exhibit a high level of anxiety and a reduction in hippocampal brain-derived neurotrophic factor (BDNF) levels [29]. BDNF is known to be related to anxiety as well as cognitive functions [30]. BDNF exerts its effect by binding to both full-length (TrkB.FL) and truncated (TrkB.T1) isoforms of the TrkB receptor. TrkB.T1 lacks the kinase domain required for the classical signal transduction pathway and has an opposing activity to that of TrkB.FL [31], causing neuronal cell death and inhibiting cell regeneration and repair. Upregulation of TrkB.T1 has been shown in numerous pathological conditions, such as cerebral ischemia [32], neuropathic pain [33], Alzheimer’s disease [34] as well as in aging [35]. Interestingly, Sirtuin 1 (Sirt1), a promising regulator of longevity [36,37,38] and a senescence marker [39], has been implicated in BDNF/TrkB signaling [40,41]. Sirt1 has two isoforms: the authentic 110 kD Sirt1 protein and a smaller 50 kD SIRT1 protein. We recently showed that old female mice exhibiting age-related cognitive decline had a larger amount of the 50 kD Sirt1 [38]. However, the long-term effect of UCMS on Sirt1 levels and the balance between TrkB isoforms has not been thoroughly investigated. 

Here, we used a model of unpredictable mild chronic stress in order to explore the harmful effects of unpredictable chronic stress on adolescents and examine its possible long-term effect on cognition. Importantly, we focus on two mechanisms of alternative splicing (AS)—of Sirt1 protein and TrkB receptor—that may be affected by UCMS exposure during adolescence. We found that stress in adolescence caused impaired cognitive behavior that is associated with an imbalance of TrkB and Sirt1 in the hippocampus and cortex, which may be related to cellular senescence. 

## 2. Results

### 2.1. Exposure to UCMS during Adolescence Shows Long-Term Effect on Anxiety-like Behavior in Both Males and Females

To evaluate the immediate, late, and long-term effect of chronic mild stress during adolescence on anxiety-like behavior, 1-month-old mice were exposed to the UCMS procedure for 4 weeks and then tested for anxiety-like behavior as described in the Methods. 

**Figure 1:**
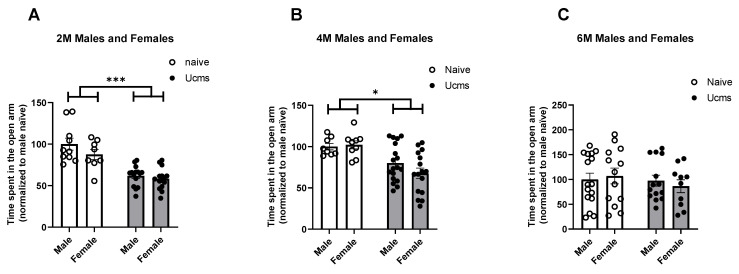
Stressed mice exhibit anxiety-like behavior in the EPM. Male and female stressed mice present higher levels of anxiety at the age of two (**A**) and four (**B**) months, which is manifested in spending significantly less time in the open arm of the EPM compared to naïve mice (*p* (Males UCMS vs. naive, 2M) < 0.0001, *p* (Females UCMS vs. naive, 2M) = 0.0001, *p* (Males UCMS vs. naive, 4M) = 0.0294, *p* (Females UCMS vs. naive, 4M) = 0.0003. At the age of six months, no significant differences in anxiety-like behavior between stressed and naïve mice were found (**A**) n(Male 2M naïve) = 10, n(Male 2M UCMS) = 15; n(Female 2M naïve) = 8, n(Female 2M UCMS) = 15. (**B**) n(Male 4M naïve) = 8, n(Male 4M UCMS) = 18. n(Female 4M naïve) = 9, n(Female 4M UCMS) = 16. (**C**) n(Male 6M naïve) = 16, n(Male 6M UCMS) = 14. n(Female 6M naïve) = 13, n(Female 6M UCMS) = 10. Data are presented as mean ± SEM and scatter dot plot. *** indicates *p* < 0.0001, * indicates *p* < 0.05.

In the Elevated Plus Maze (EPM, see Methods section for details) test (Figure 1), male and female stressed mice presented higher levels of anxiety-like behavior at the age of two months (Figure 1A; F (1, 44) = 49.63, *p* < 0.0001) and four months (Figure 1B; F (1, 47) = 19.15, *p* < 0.0001), which manifested in a significant decrease in the time spent in the open arm of the EPM compared to naïve mice. LSD post hoc *p* values: (*p* (Males UCMS vs. naive, 2M) < 0.0001, *p* (Females UCMS vs. naive, 2M) = 0.0001, *p* (Males UCMS vs. naive, 4M) = 0.0294, *p* (Females UCMS vs. naive, 4M) = 0.0003). At the age of six months, no significant differences were found between stressed and naïve mice of both sexes (Figure 1C). There was no gender effect in either age.

**Figure 2:**
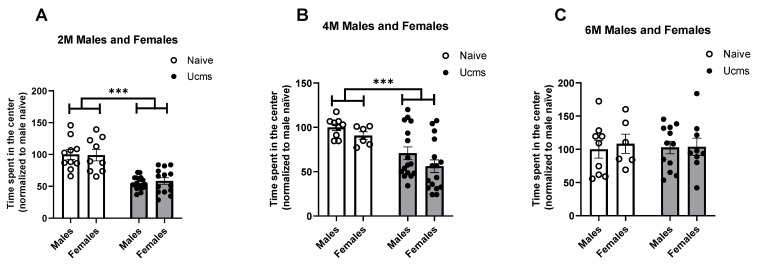
Stressed mice exhibit increased anxiety-like behavior in the OFT. Male and female stressed mice present higher levels of anxiety at the age of two ((**A**), F (1, 46) = 56.24, *p* < 0.0001) and four months ((**B**), F (1, 43) = 16.55, *p* = 0.0002), which manifested in spending significantly less time in the center of the OF compared to naïve mice At the age of six months, no significant differences were found between stressed and naïve mice (**A**) Male 2M n(naïve) = 10, n(UCMS) = 17. Female 2M, n(naïve) = 9, n(UCMS) = 14) (**B**) Male 4M t(df = 23) = 2.92, n(naïve) = 9, n(UCMS) = 16. Female 4M n(naïve) = 6, n(UCMS) = 16). (**C**) Male 6M n(naïve) = 9, n(UCMS) = 12. Female 6M n(naïve) = 6, n(UCMS) = 9). Data are presented as mean ± SEM and scatter dot plot. *** indicates *p* < 0.0002.

In the Open Field Test (OFT, see Methods for details) (Figure 2), male and female stressed mice presented higher levels of anxiety-like behavior at the age of two months (Figure 2A, F (1, 46) = 56.24, *p* < 0.0001) and four months (Figure 2B, F (1, 43) = 16.55, *p* = 0.0002), which manifested as a significant decrease in the time spent in the center of the OF compared to naïve mice. LSD post hoc *p* values: (*p* (Males UCMS vs. naive, 2M) < 0.0001, *p* (Females UCMS vs. naive, 2M) < 0.0001, *p* (Males UCMS vs. naive, 4M) *p* = 0.0068, *p* (Females UCMS vs. naive, 4M) = 0.0056). At the age of six months, no significant differences were found between stressed and naïve mice of both sexes (Figure 1C). There was no gender effect in either age.

Of note, no differences were found in the activity of male and female mice among the different groups at each age (see Supplementary Figure S1).

### 2.2. Exposure to UCMS during Adolescence Impaired Cognition Later in Life

To evaluate the long-term effect of chronic mild stress during adolescence on spatial memory and cognition, we used a Y-maze. Mice were tested 2 and 4 months after the exposure (at the age of 4 and 6 months, respectively). 

**Figure 3:**
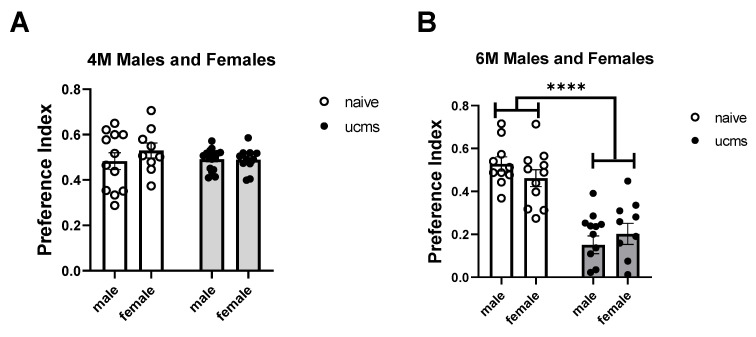
Stressed mice exhibit spatial memory impairment in the Y-maze. At the age of 4 months (**A**), male and female stressed mice show no significant differences in their preference index of the Y-maze compared to naïve mice. At the age of 6 months (**B**), both male and female stressed mice spent significantly less time in the novel arm of the Y-maze compared to naïve mice, resulting in decreased preference index. (*p* (Males UCMS vs. naive) < 0.0001, *p* (Females UCMS vs. naive) < 0.0001)). Data are presented as mean ± SEM and scatter dot plot. **** indicates *p* < 0.0001.

We discovered that both stressed male and female mice exhibited spatial memory impairment in the Y-maze at the age of 6 months (F (1, 40) = 58.53, *p* < 0.0001) but not at the age of 4 months (Figure 3). Stressed mice spent significantly less time in the novel arm of the Y-maze compared to naïve mice. There was no gender effect in either age.

(*p* (Males UCMS vs. naive) < 0.0001, *p* (Females UCMS vs. naive) < 0.0001): (A) Male 4M n(naïve) = 12, n(UCMS) = 16. Female 4M n(naïve) = 9, n(UCMS) = 11). (B) Male 6M n(naïve) = 10, n(UCMS) = 13. Female 6M, n(naïve) = 11, n(UCMS) = 10).

### 2.3. Imbalance in SIRT1 Alternative Splicing in Response to UCMS

We assayed the levels of two known isoforms of Sirt1-50 kD and 110 kD using both WB and qPCR in the hippocampus and cortex of male and female mice and assessed the 50/110 ratio in order to examine the involvement of this pathway in the long-term deleterious effects of chronic mild stress exposure during adolescence. We were not able to detect the Sirt1-110 kD protein in males and thus present only the result in females (see the Supplementary File for the male results).

**Figure 4:**
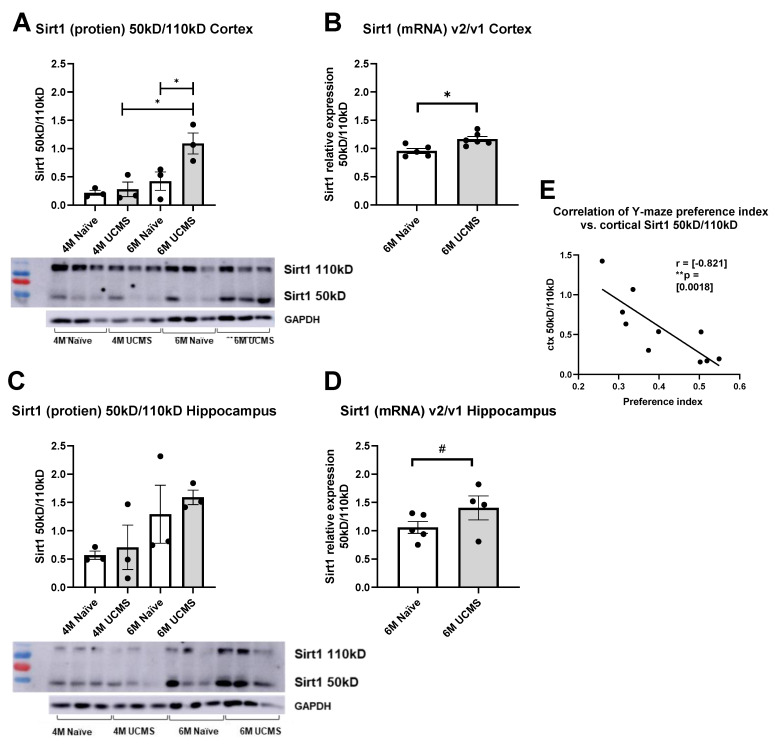
Stressed female mice show an imbalance in sirt1 50 kD/110 kD ratio in specific brain regions. Representative Western blot images of each region are shown above. (**A**) Cortical sirt1 50 kD/110 kD protein expression ratio. A significant increase in sirt1 50 kD/110 kD ratio among 6-month-old stressed female mice compared to naïve age-matched mice (* *p* = (0.0406), *n* = 3). A significant increase in sirt1 50 kD/110 kD ratio between 4 and 6 months old UCMS female mice (* *p* = (0.0151), *n* = 3). (**A**) Two-way ANOVA analysis of the effect of age and UCMS paradigm on sirt1 50 kD/110 kD ratio showed a statistically significant simple main effect of age (** *p* = (0.0069)) and of the UCMS paradigm (* *p* = (0.0322)). (**B**) Cortical sirt1 short/long mRNA expression ratio increased in the cortex of stressed mice (*p* = 0.009, t = 1.552, df = 7). (**C**) Hippocampal sirt1 50 kD/110 kD protein expression ratio. Stressed female mice show no significant differences in sirt1 50 kD/110 kD ratio hippocampal levels compared to naïve age-matched mice (*p* > 0.05, *n* = 3). (**D**) Stressed female mice show a trend toward an increase in the sirt1 short/long mRNA expression ratio in the hippocampus (*p* = 0.08). (**E**) Correlation of cortical sirt1 50 kD/110 kD vs. Y-maze preference index. A Pearson correlation coefficient was computed to assess the linear relationship between cortical sirt1 50 kD/110 kD ratio and Y-maze Preference Index scores. There was a negative correlation between the two variables, r(8) = (−0.821), ** *p* = (0.0018).

We analyzed the Sirt1 50 kD/110 kD ratio in the cortex of female mice at the age of 4 months (2 months after the exposure to UCMS using WB (Figure 4A) and at the age of 6 months after the exposure to UCMS using WB (Figure 4C)). We found a significant change in Sirt1 50 kD/110 kD ratio in the cortex of mice between the age of 4 and 6 months (*p* = 0.0036) and between stressed and naïve mice at 6 months (*p* = 0.01) compared with naïve mice, indicating a tendency toward the 50 kD isoform after stress. There was no difference in the hippocampus (Figure 4C). Interestingly, the elevation of Sirt1 50 kD/100kD ratio in the cortex following UCMS during adolescence correlated with the impairment in cognition (Figure 4E). We also analyzed Sirt1 mRNA short/long-expression ratio in the cortex of female mice at the age of 6 months (Figure 4B) and in the hippocampus (Figure 4D). We found a significant change in Sirt1 short/long mRNA ratio in the cortex of mice between stressed and naïve mice (*p* = 0.009) and a trend in the hippocampus (*p* = 0.08).

### 2.4. Imbalance in TrkB Receptor Alternative Splicing in Response to UCMS

**Figure 5:**
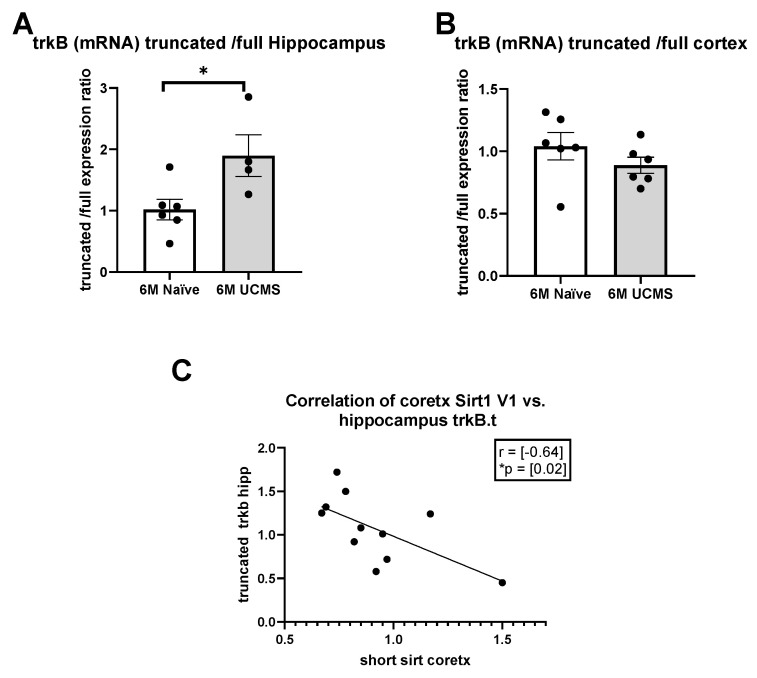
Stressed female mice show an imbalance in sirt1 Trkb.T1/TrkB.full ratio in specific brain regions. A significant increase in TrkB.t1 receptor/TrkB.FL receptor among 6-month-old stressed female mice compared to naïve age-matched mice in the Hipp (**A**) (* *p* = (0.03), *n* = 6), indicating a higher tendency to express TrkB.t1 over the Trk.B full receptor. There was no difference in the CTX (**B**) (* *p* = (0.07), *n* = 6). A correlation coefficient was computed to assess the linear relationship between the long sirt1 in the PFC and the TrkB.t receptor in the hippocampus (**C**). There was a negative correlation between the two variables, r(11) = (−0.64), * *p* = (0.02).

We assayed the levels of two known isoforms of TrkB receptor—TrkB full receptor and TrkB truncated receptor using qPCR in the hippocampus and cortex of female mice and assessed the short/full ratio in order to examine the involvement of this pathway in the long-term deleterious effects of chronic mild stress exposure during adolescence. We saw a significant increase in TrkB.t1/TrkB.full receptor among 6-month-old stressed female mice compared to naïve age-matched mice in the hippocampus (*p* = 0.03), indicating a higher tendency to express TrkB.t1 over the Trk.B full receptor (Figure 5A). There was no difference in the CTX (Figure 5B). As we found a significant change in TrkB.t/TrkB.Full receptor ratio in the hippocampus and a significant change in short/long sirt1 ratio in the PFC, a correlation coefficient was computed between the beneficial long sirt1 in the cortex and the maladaptive TrkB.t in the hippocampus (Figure 5C). There was a negative correlation between the two variables, r(11) = (−0.64), * *p* = (0.02).

## 3. Discussion

This study demonstrates that exposure to unpredictable chronic mild stress during adolescence has long-term effects on cognition and anxiety-like behavior. These behavioral changes were accompanied by a significant imbalance between the two isoforms of both the Sirt1 protein and the TrkB receptor. Male and female mice exposed to unpredictable stress during the adolescence period responded with elevated stress response in the behavioral tests immediately after the exposure, as expected (as discussed in the introduction [29,42]), as well as two months after the exposure. Interestingly, 4 months after the UCMS, although the anxiety-like behavior was no longer evident, male and female mice exhibited significant spatial memory impairment in the Y-maze. This suggests a stream of events that take place following stress at a critical time point in adolescence that may affect cognition in adulthood. 

Uncertainty and unpredictability regarding the future are important factors in the generation of stress and anxiety [43]. The UCMS paradigm [28] for animal models, used in this research, exploits exactly these vulnerabilities. The stressors are administered randomly, and the stress sequence changes every week in order to make the stress procedure unpredictable. The inability of the animal to predict the stressor and the loss of control is critical to the outcome of the UCMS paradigm [44]. Furthermore, young people seem to be especially sensitive to this uncertainty [45]. The adolescence period is a sensitive time for brain development [22,24,46]. Although massive neurological changes make this period especially sensitive to stress-related changes [23], most studies on the long-term effect of early exposure to stress focus on early childhood [20]. Few studies did attempt to explore this delicate period. For example, Sterlemann and colleagues [47,48] demonstrated that chronic social stress during adolescence had an immediate effect on behavioral performance and neuroendocrine (corticosterone and CRH) levels, and some of the effects (specifically anxiety-related behavior and spatial memory) were also evident 12 months after the exposure to social stress. These results are intriguing, but they are limited to predictable social stress only, and importantly, because the mice were examined at the age of 15 months, natural aging effects might have been an intervening factor. For this reason, we examined adult, but not old mice, to see if the exposure to unpredictable chronic mild stress during adolescence may be a risk factor for cognitive decline. Indeed, in our study, 1-month-old adolescent mice exposed for a month to unpredictable stress, and examined 4 months later, during adulthood, exhibited cognitive deficits in spatial memory. 

In our results, there was no behavioral difference in the stress response of male and female mice. This contrasts with previous reports indicating a different stress response between males and females [21,26]. However, when looking at the biological implications of stress exposure, we did find a gender effect. The cognitive decline observed was accompanied by significant changes in the alternative splicing of the TrkB receptor and Sirt1 protein in females. Alternative splicing produces multiple protein isoforms through using different sets of exons in more than 90% of the human protein-coding genes [49]. AS is involved in many normal and pathological biological processes, such as neurogenesis and brain development [50], stress response [51], and also in senescence and aging [52,53].

Sirt1 is one of the most promising regulators of longevity—the level of Sirt1 was found to decrease during aging [36], while mice with brain-specific transgenic over-expression of Sirt1 have an extended lifespan [37]. Furthermore, recent findings suggest that adult mice exposed to chronic stress show lower Sirt1 levels and impaired cognitive functions [54].

Sirt1 undergoes alternative splicing to generate a shorter isoform. This shorter isoform has only minimal deacetylase activity and exhibits distinct stress sensitivity and stability compared to full-length Sirt1 [55]. Indeed, we showed that a single injection of an ultra-light dose of THC (ULD-THC) ameliorated the cognitive functioning of old mice and elevated the level in the brain of Sirt1, thus showing a rejuvenating effect of ULD-THC on the brain. Interestingly, old mice, exhibiting age-related cognitive decline, had a larger amount of the 50 kD SIRT1 isoform compared to the longer, 100 kD Sirt1 isoform [38]. 

In accordance with these findings, we show here that 4 months after exposure to chronic unpredictable mild stress, there was an increase in short/long Sirt1 ratio both in mRNA expression and in protein level, indicating an increase in Sirt1 V2 transcript variant and 50 kD protein levels in UCMS-exposed mice only. This elevation of Sirt1 50 kD/100kD ratio in the hippocampus following UCMS during adolescence correlated with the impairment in cognition.

Loss of function of Sirt1 down-regulates the expression of cAMP response binding protein (CREB), impacting the neurotropic system, synaptic plasticity and BDNF expression [56]. Brain-derived neurotrophic factor (BDNF) is produced by astrocytes [57] and neurons [58] and is a crucial mediator of neuronal plasticity [59] and neurogenesis [60,61]. Patients with anxiety disorder have lower central and peripheral levels of BDNF [62,63]. In rodents, BDNF levels in the hippocampus and prefrontal cortex (PFC) were decreased following chronic stress manipulations and increased following chronic treatment with several different antidepressants [64,65,66,67,68]. BDNF exerts its effect by binding to both full-length (TrkB.FL) and truncated (TrkB.T1) isoforms of the TrkB receptor. TrkB.T1 lacks the kinase domain required for the classical signal transduction pathway and has an opposing activity to that of TrkB.FL [31], causing neuronal cell death and inhibiting cell regeneration and repair. The TrkB.FL/Trk.T1 ratio has been shown to have clinical importance. In schizophrenia patients, this ratio had a predictive value, as a lower TrkB.FL/TrkB.T1 ratio in the periphery (indicating lower expression of the full receptor and higher truncated expression) was associated with worse clinical response to antipsychotic [69].

Our data suggested that UCMS changes the balance between the full and the truncated TrkB receptor so that mice exposed to chronic mild stress exhibit a higher tendency to express the truncated isoform. Furthermore, up-regulation of the beneficial full-length Sirt1 isoform in the cortex (usually seen in young non-stressed animals) was negatively correlated with the expression of the maladaptive TrkB.T1 receptor in the hippocampus.

Thus, a possible explanation for our results is that the cognitive impairment manifested 4 months after the exposure to chronic stress, at the age of 6 months, may be related to accelerated senescence-related alternative splicing of Sirt1 in the cortex and TrkB in the hippocampus [70]. One potential pathway may be the failure of astrocytes to support neuronal cells activity, as previously suggested [71].

It was previously shown that chronic stress could lead to anxiety-like behavior [42,65,72]. Blackburn and colleagues showed in humans that chronic stress resulted in cellular senescence [12,73]. Meanwhile, Montaron et al. [74] showed in rodents that lowering corticosterone secretion from midlife on increases neurogenesis and prevents the emergence of age-related impairments in spatial memory. The ‘free radical theory of aging [75] suggests that aging is a result of the accumulation of reactive oxygen species (ROS) that attacks the DNA. These age-related changes are accompanied by cellular senescence, a permanent state of cell cycle arrest, which contributes to aging by depleting the tissues of cells that are able to proliferate [76], as well as with telomere shortening. Telomeres are the terminal regions of chromosomes that are required for chromosomal stability and essential for correct DNA replication, which shorten with each cell division [12]. Stress is one of the known triggers for cellular senescence [73]. Various intrinsic and extrinsic insults can cause stress-induced premature senescence, such as oxidative and genotoxic stress, mitochondrial dysfunction or chemotherapeutic agent [77]. Here, we suggest for the first time, to the best of our knowledge, that exposure to unpredictable chronic mild stress during adolescence may contribute to cognitive impairment via cellular senescence mechanisms. Of note, it was suggested that stress in adolescence might negatively affect hippocampal neurogenesis and therefore impair cognition [78]. Once cellular senescence appears, it may be non-reversible and therefore may not require further stress exposure to cause impaired cognition. 

We discovered that UCMS yielded a significant imbalance in the alternative splicing of Sirt1 protein and TrkB receptor, with a negative correlation between the expression of the full-length Sirt1 and the truncated TrkB. Thus, we postulate that as the level of 110 kD Sirt1 diminishes during aging, TrkB.T1 is up-regulated and that this process also happens after exposure to chronic stress. More research is needed in order to further explore this possibility by examining other types of age-related cognitive deficiencies, such as executive functions or non-spatial long-term memory as well as investigating the long-term effect of stress on other cellular-senescence mechanisms.

Surprisingly, we detected the 50 kD Sirt1 isoform only in female mice. This may be related to gender differences in age-related molecular pathways. In humans, when examining serum from aged men and women, only women showed age-related alternation in Sirt1 activity [79]. Importantly, Sirt1 expression was susceptible to lifestyle factors only in women [80]. Sexual dimorphism in Sirt1 expression was also shown in aged mice [81]. In fact, old female mice were more susceptible to LPS-induced depression, and this was accompanied by different Sirt1 activation between males and females. The authors suggested that Sirt1 may be regulated by estrogen levels [82]. Thus, our chronic stress manipulation might have had a different biological effect on the females, resulting in different Sirt1 isoform expressions. Additional experiments are needed to understand further the differences between the genders. 

To conclude, our results demonstrate a long-term cognitive deleterious effect of chronic unpredictable mild stress exposure during adolescence. This effect was evident even though anxiety-like behavior was no longer detectable at that time. Furthermore, we discovered that stress in adolescence affects male and female mice and is linked to the expression of Sirt1 and TrkB. Further study of the relations between stress and senescence markers might increase our understanding regarding cognitive impairments affiliated with stress. It was previously suggesting that the enriched environment in the adolescent stages in animals can reduce stress-related cognitive decline [83,84].

Our paper is in line with other publications that emphasize the importance of a sense of control in adolescents in order to increase their well-being and minimize the effect of the unpredictable nature of this situation on later cognition.

## 4. Materials and Methods

### 4.1. Experimental Design

Three cohorts of 1-month-old male and female ICR mice were exposed to the UCMS paradigm (stress group) or received standard care (naïve group) for 4 weeks. Each cohort underwent behavioral examinations at different time points: (i) at the age of 2 months, immediately after the UCMS; (ii) at the age of 4 months, 2 months after the UCMS; and (iii) at the age of 6 months, 4 months after the UCMS. Each mouse was examined in all 3 behavioral tests, one each day. One day after the last behavioral test, mice were sacrificed and brain tissue was collected. 

### 4.2. Animals

All experiments were performed on male and female C57BL/6J mice (Jackson laboratories). Mice were kept in the vivarium of the Psychobiology laboratory of the Open University located at Hadassah Medical Center, Jerusalem on a reversed 12 h light/dark cycle. All experiments were performed under red light during the dark phase (7:00–19:00). Mice were given ad libitum access to food and water. The care and experimental use of all animals were performed in accordance with the Open University guidelines. All experiments were approved by the university’s animal care committee (approval number IL-19-4-178, 04/2019)

### 4.3. Unpredictable Chronic Mild Stress (UCMS) Manipulation

The procedure was performed during adolescence, starting at the age of 30 days and lasting for four weeks, as previously described by us [42]. Mice were subjected to unpredictable stress using the following stressors: placement in an empty cage with 1 cm of water at the bottom, light/dark cycle inversion, placing the mice in cages with wet sawdust, tilting the cages at 30 degrees, inducing social stress by placing mice in soiled cages of other mice and restraining the mice. The mice were exposed to each stressor for four hours (excluding the light/dark cycle inversion, which lasted for 48 h) at different times of the day and at random. 

### 4.4. Open Field Test (OFT)

Anxiety-like behaviors were assessed using the OFT as previously described by us [85]. This test reflects the conflict between the instinctive fear that mice have from exposed areas, such as the central area of the arena, versus their curiosity and tendency to explore new environments. When anxious, mice will tend to prefer staying close to the walls. The open field consisted of an empty arena (40 × 40 × 40 cm). Mice were placed in the arena for a total of 5 min. Anxiety-like behavior was measured by the time spent near the walls in the peripheral area. Data were normalized to naïve males.

The OFT was also used to assess motor function. The total distance moved in the arena was measured (in cm). The activity of each mouse was normalized compared to naïve male mice (see Supplementary Figure S1).

### 4.5. Elevated plus Maze (EPM)

Anxiety-like behaviors were also assessed using the EPM, as previously described by us [85]. This task is based on the instinctive tendency of mice to prefer closed places and avoid open and elevated places. The apparatus is situated 40 cm above the floor. It consisted of a plus-shaped maze with two black plastic closed arms and two opposite open arms. The mouse was placed in the center of the EPM for 5 min. Anxiety-like behavior was determined by the time the animal spent in the open, unprotected arm of the maze. Data were normalized to naïve-males.

### 4.6. Y-Maze 

Short-term spatial memory was assessed using the Y-maze (YM) assay. This is based on the mice’s preference to explore a new environment [38]. The maze consisted of 3 identical arms in a Y shape. Each arm is 30 cm long and separated by 120° angles, with each arm presenting different visual cues. During the first session, the mouse was placed at the distal edge of the start arm with 1 of the 2 remaining arms blocked and left to explore the maze for 5 min. The mouse was then removed to its home cage and reintroduced to the start arm 2 min later for 2 min with the 2 remaining arms open. The time the mouse explored the 2 arms was recorded. Preference Index (PI) was calculated as follows: (time in new arm-time in the familiar arm)/(time in new arm + time in the familiar arm).

All behavioral assays were recorded and analyzed using the Biobserve software.

### 4.7. Western Blot (WB)

In order to assess the long-term effect of stress on Sirt1 protein changes, we performed WB at the age of 4 and 6 months. A section was collected from the cortex and hippocampus from brain frozen section using the cryostat, as cited before ([71]. Protein concentrations were determined using Bradford reagent (Bio-Rad Laboratories, Hercules, CA, USA), using bovine serum albumin as a standard. For SDS-PAGE separations, 10% polyacrylamide gels were used. Samples were transferred to nitrocellulose membranes. Membranes were blocked with 5% skim milk block buffer for 1 h, washed with 0.05% Tween in TBS (TBST), and probed with mouse anti sirt1 (1:1000, abcam, ab-110304) overnight at 4 °C. The membrane was washed three times for 10 min and incubated with secondary antibodies (1:10,000, Jackson laboratories, 115-035-003) for 1 h at room temperature. Protein bands were detected using the Amersham Imager 600 (GE Healthcare, Chicago, IL, USA). The membranes were washed three times for 10 min with TBST and probed with mouse anti GAPDH (1:10,000, Millipore, Cat#MAB374). The membranes were then washed and incubated with a secondary antibody. Band intensity was measured using the Amersham Imager 600, and the relative band intensity was determined using Image-J.

### 4.8. Real-Time PCR

Total RNA was extracted from the hippocampus and cortex using the PureLink RNA Mini Kit (Rhenium, Modi’in-Maccabim-Re’ut, Israel) according to the manufacturer’s instructions. SYBR Green real-time PCR primers were purchased from Agentek Israel. RT-PCR was performed with primers specific for full tropomyosin receptor kinase B (TrkB.full) and truncated tropomyosin receptor kinase B TrkB.T1, Sirtuin.v1 and Sirtuin.v2 (Agantek, Yakum, Israel) using the Magnetic Induction Cycler (Mic) PCR Machine (biomolecular systems). Delta–delta values were calculated compared to GADPH gene. Changes in RNA alternative splicing were examined at the age of 6 months only at the time when protein changes were found to be significant. 

### 4.9. Statistical Analysis

Data analyses were carried out using GraphPad Prism 8. Statistical significance was measured with Student’s t-test (between two groups) and ANOVA (analysis of variance) with Fisher’s LSD comparison test for the comparison of 4 groups. Normality was examined for each data set before carrying out the parametric tests. Linear correlation was determined by calculating Pearson’s correlation coefficients. *p*-values of less than 0.05 were considered statistically significant.

## Supplementary Materials

The following supporting information can be downloaded at: https://www.mdpi.com/article/10.3390/ijms23094945/s1.
