# Supplementary material for: Imbalance in Sirt1 Alternative Splicing in Response to Chronic Stress during the Adolescence Period in Female Mice
Source: Int J Mol Sci. 2022 Apr 29;23(9):4945. doi: 10.3390/ijms23094945 (PMC9104080; doi:10.3390/ijms23094945)

Supplementary file

No difference in Motor functions between naïve and UCMS-exposed mice

Motor functions were evaluated in the open field test at each time point. No gender or group changes were observed (Figure S1). Stressed mice are as active as naïve mice in the OFT throughout the experiment. Two-way Anova yielded no significant differences in the activity of male and female mice among the different groups at the age of two (A), four (B) and six months old (C)

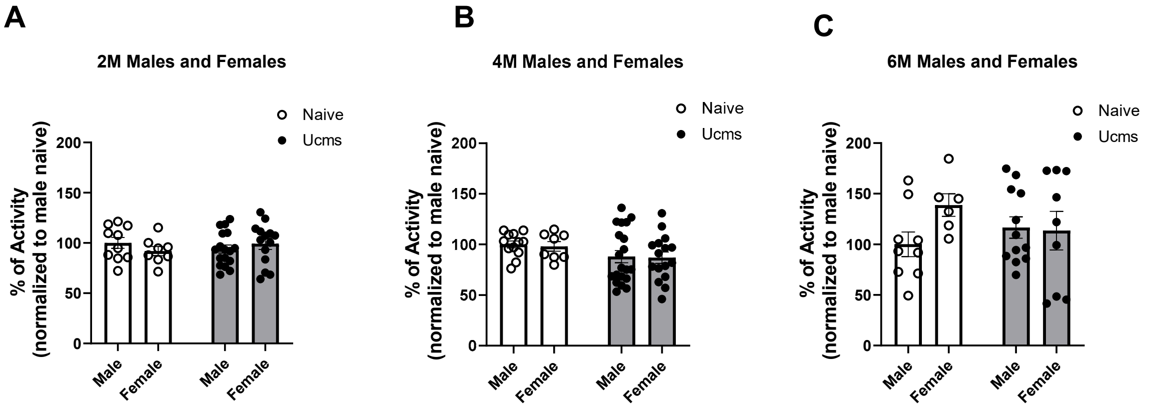

Figure S1: Stressed mice are as active as naïve mice in the OFT throughout the experiment. No differences were found in the activity of male and female mice among the different groups at the age of two (A), four (B) and six months old (C) [(M, 2months, naïve)=10, n(M, 2months UCMS)=17. n(F,2months ,naïve)=8, n(F,2months UCMS)=14), n(M,4months ,naïve)=12, n(M,4months ,UCMS)=20. n((F,4months ,naïve)=8, n((F,4months ,UCMS)=16), (n(M,6months ,naïve)=9, n(M,6months ,UCMS)=12. p(F,6months)>0.05, n(naïve)=6, n(UCMS)=9). Data is presented as mean ± SEM and scatter dot blot.

Sirt1 110kD changes after exposure to UCMS in male mice

We were not able to detect Sirt 50kD either in the cortex or the hippocampus of male mice (Figure S2). In the cortex, Sirt 110kD was significantly decreased among 4 months old stressed male mice, compared to naïve age-matched mice and there was also a significant decrease in sirt1 levels among 6 months old naïve male mice, compared to 4 months old naïve mice (Figure S2A). A two-way ANOVA analysis of the effect of age and UCMS paradigm on sirt1 protein expression showed a statistically significant interaction between the effects of age and paradigm (F (1,8)= [5.660], *p = [0.0446]). Simple main effects analysis showed a statistically significant effect of age on sirt1 protein expression (**p = [0.0048]), and of the UCMS paradigm on sirt1 protein expression (*p = [0.0151]). There was no difference in the Hippocampal sirt1 110kD_protein expression levels between the groups (Figure S2B).

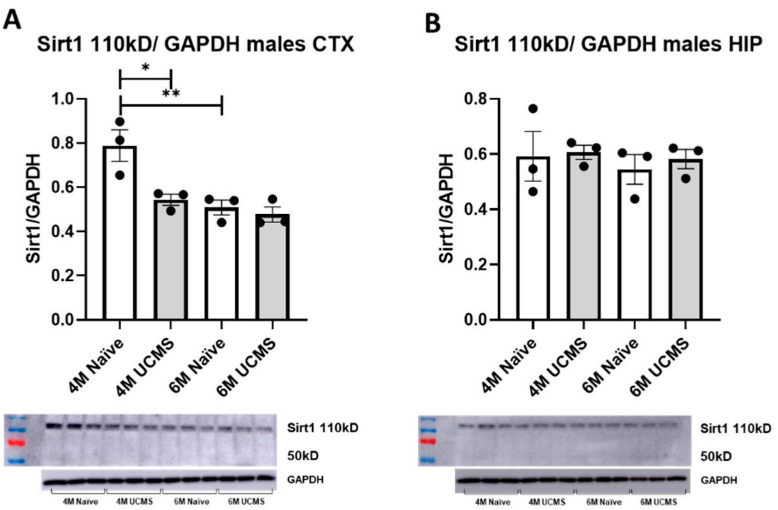

Figure S2: Stressed male mice show a decrease in cortical sirt1 110kD levels. Representative Western blot images of each region are shown above. (A) Cortex sirt1 protein expression. a significant decrease in sirt1 levels among 4 months old stressed male mice, compared to naïve age-matched mice (*p = [0.0201], n=3). a significant decrease in sirt1 levels among 6 months old naïve male mice, compared to 4 months old naïve mice (**p = [0.0096], n=3). A two-way ANOVA analysis of the effect of age and UCMS paradigm on sirt1 protein expression showed a statistically significant interaction between the effects of age and paradigm (F (1,8)= [5.660], *p = [0.0446]). Simple main effects analysis showed a statistically significant effect of age on sirt1 protein expression (**p = [0.0048]), and of the UCMS paradigm on sirt1 protein expression (*p = [0.0151]). (B) Hippocampal sirt1 protein expression. stressed male mice show no significant differences in sirt1 hippocampal levels, compared to naïve age-matched mice (p>0.05, n=3).

<u>Sirt-1 110kD and 50 kD protein levels changes differently in the hippocampus and cortex of female mice after exposure to UCMS</u>

In the cortex, female mice exhibited downregulation of sirt1 110kD protein expression between 4 and 6 months age (Figure S2A ;p = [0.0022]). There was no difference in the expression of 110kD in the hippocampus (Figure S2C). Cortical (Figure S2B) and hippocampal (Figure S2D) levels of sirt1 50kD protein expression did not differ between the group.

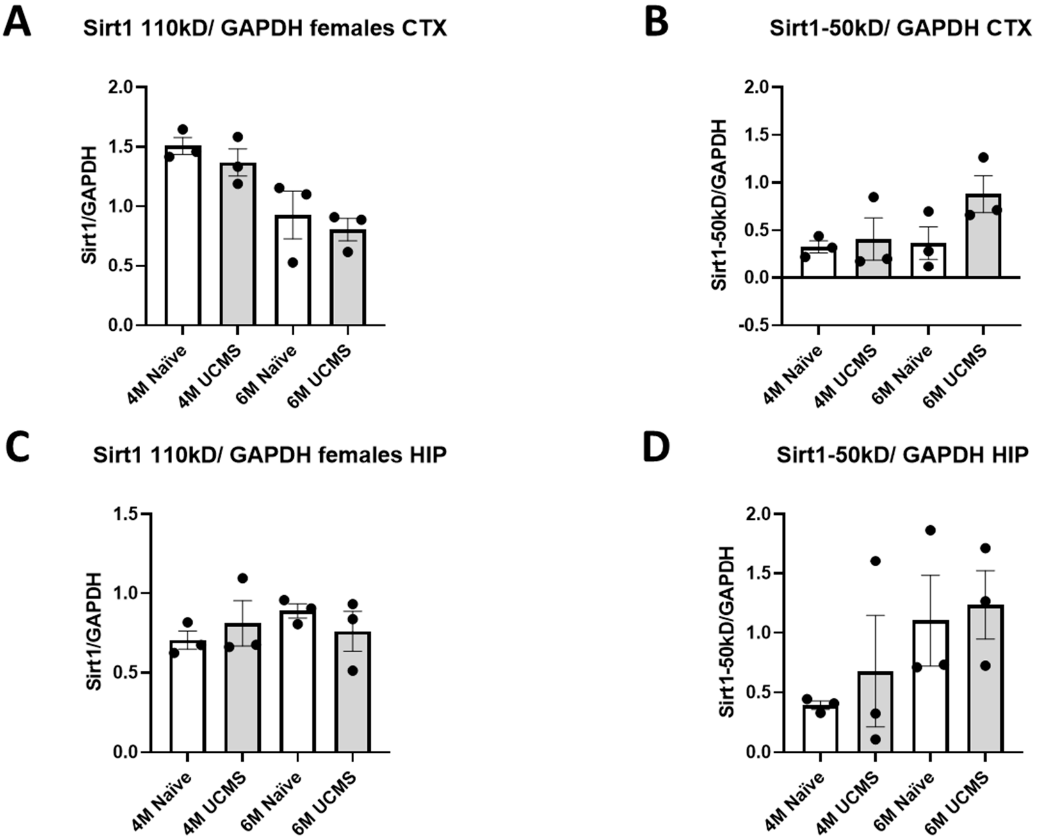

Figure S3: Female mice sirt1 levels in specific brain regions. (A) Cortical sirt1 110kD protein expression. Simple main effects analysis showed a statistically significant effect of age on sirt1 protein expression (**p = [0.0022]). (B) Cortical sirt1 50kD protein expression. (C) Hippocampal sirt1 110kD protein expression. (D) Hippocampal sirt1 50kD protein expression.

Sirt-1 v1 and v2 mRNA relative expression in the hippocampus and cortex in 6 months old females

While there was a difference in the ratio between the short (v2) and the long (v1) Sirt-1 mRNA expression level (figure 4 in manuscript), there was no difference between the UCMS and naïve mice in each of the isoform individually (figure S4).

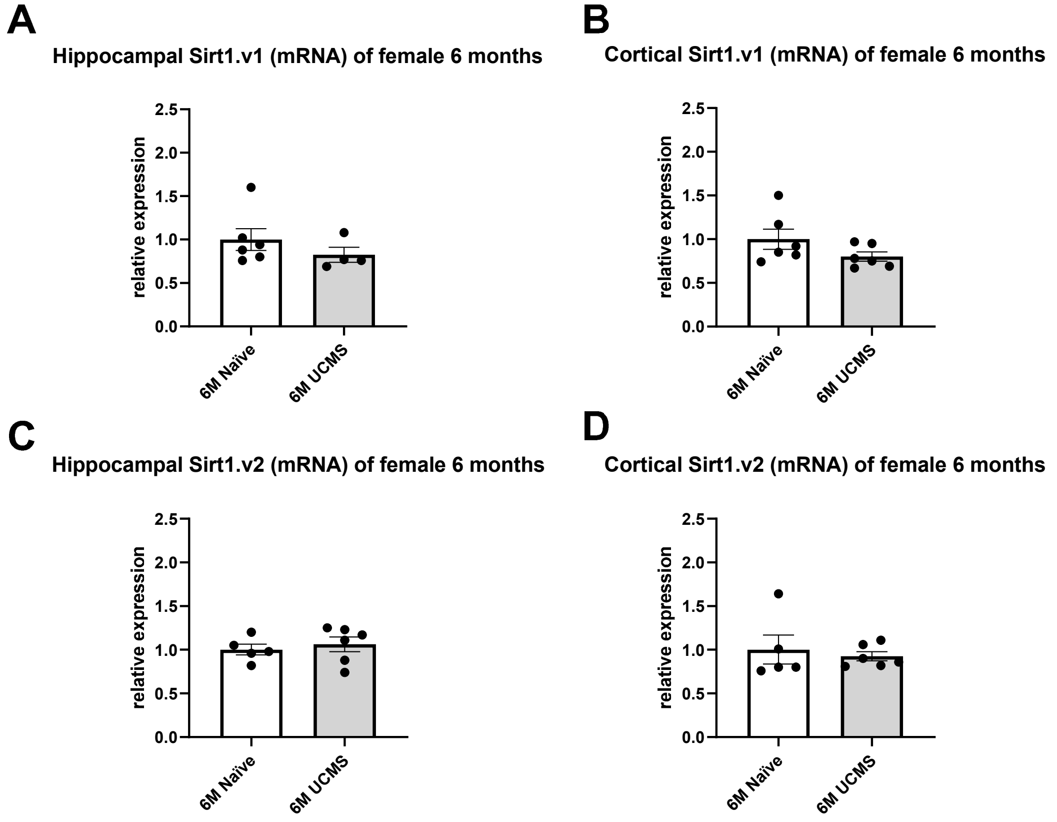

Figure S4: 6 months old female mice sirt1 mRNA expression levels in specific brain regions. (A) Hippocampal sirt1.V1 mRNA relative expression. (B) Cortical sirt1.V1 mRNA relative expression. (C) Hippocampal sirt1.V2 mRNA relative expression. (D) Cortical sirt1.V2 mRNA relative expression.

TrkB.full and TrkB.t mRNA relative expression in the hippocampus and cortex in 6 months old females

While there was a difference in the ratio between the TrkB.t and the TrkB.full mRNA expression level (figure 5 in manuscript), there was no difference between the UCMS and naïve mice in each of the isoform individually (figure S5).

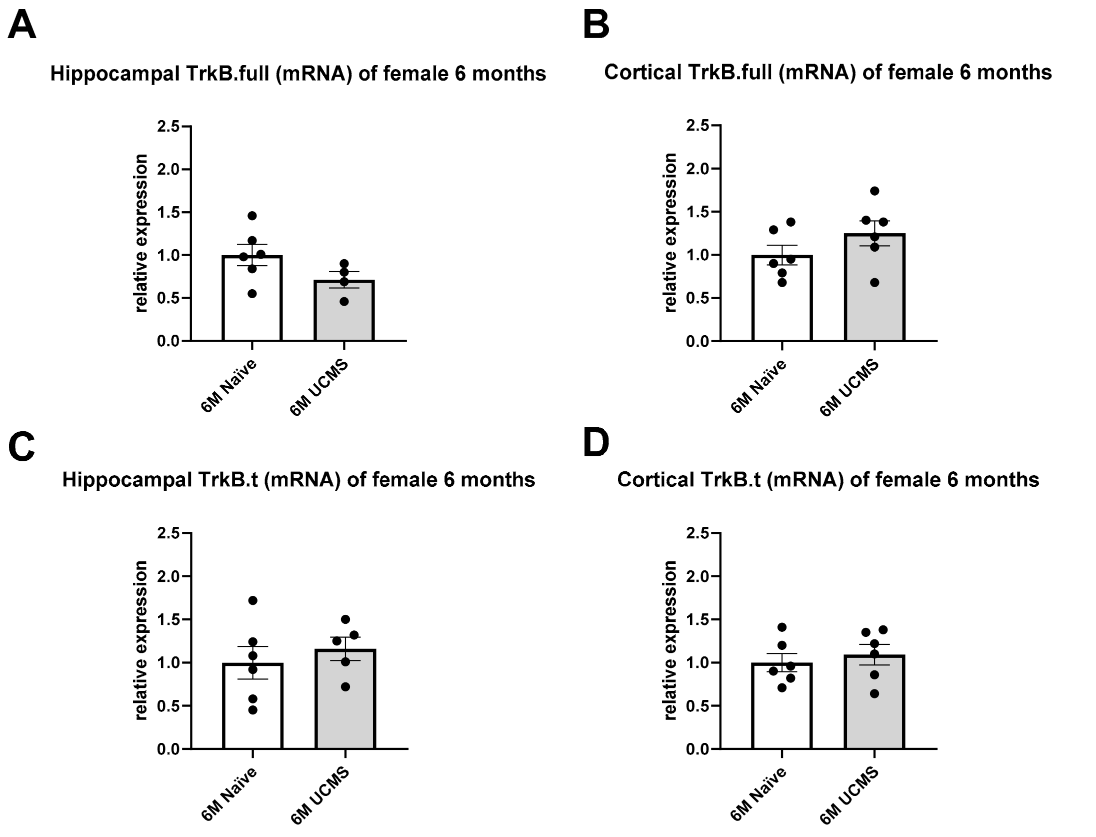

Figure S5: 6 months old female mice TrkB mRNA expression levels in specific brain regions. (A) Hippocampal TrkB.full mRNA relative expression. (B) Cortical TrkB.full mRNA relative expression. (C) Hippocampal TrkB.t mRNA relative expression. (D) Cortical TrkB.t mRNA relative expression.

Table S1 - List of primers sequences:

NAME	FORWARD	REVERSE
TRKB FULL (NTRK2 FULL)	GTCAGCTCAAGCCAGACACATTT	CTGCTCTGGGCAGAGGTTGT
TRKB T1 (NTRK2 T)	ATTCCAAGTTTGGCATGAAAGGT	GTCCCAGAGTTCAGCTCACAG
SIRT V1	TTGACCGATGGACTCCTCAC	GTCACTAGAGCTGGCGTGTG
SIRT V2	CGGCTACCGAGGTCCATATAC	AGTCAGGTGGAGGAATTGT

Figure S6 – Experimental timeline

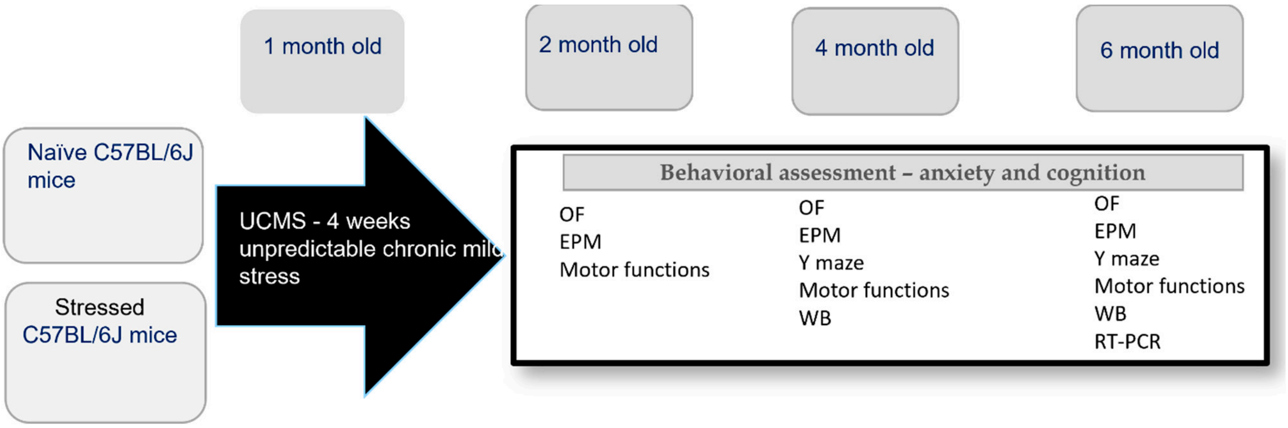

Supplement: Supplementary file 1 [file ijms-23-04945-s001.zip › ijms-1658135-supplementary.pdf]
